# Lower blood malondialdehyde is associated with past pesticide exposure: findings in Gulf War illness and healthy controls

**DOI:** 10.1186/s40779-021-00337-0

**Published:** 2021-08-17

**Authors:** Beatrice Alexandra Golomb, Sridevi Devaraj, Alexis K. Messner, Hayley Jean Koslik, Jun Hee Han, Barnabas Yik

**Affiliations:** 1 Department of Medicine, School of Medicine, University of California, San Diego, La Jolla, CA 92093 USA; 2 Department of Pathology and Immunology, Baylor College of Medicine, Houston, TX 77030 USA; 3 Swedish Medical Center, Seattle, WA 98109 USA; 4 Henry M. Jackson Foundation for the Advancement of Military Medicine, San Diego, CA 92134 USA; 5 Santa Clara Valley Medical Center, San Jose, CA 95128 USA

**Keywords:** Malondialdehyde, Oxidative stress, Free radical, Gulf War veterans, Gulf War illness, Pesticide

## Abstract

**Background:**

Malondialdehyde (MDA) is a candidate general marker of oxidative stress (OS). We sought to assess the relation of MDA to Gulf War illness (GWI) and to a variety of exposures.

**Methods:**

This is an observational study involving subjects from Southern California recruited from October 2011 to May 2014. MDA was assessed in 81 participants (41 GWI-cases, 40 controls). General and Gulf-specific exposures were elicited. MDA case–control comparison was restricted to 40 matched pairs. The potential association between MDA and exposures was assessed using regression analyses. Gulf-specific exposures were incorporated into a case-specific model.

**Results:**

Plasma MDA was significantly lower in GWI-cases than controls. Composite pesticide and fuel-solvent exposures negatively predicted MDA in the total sample, as well as in the analyses that included either GWI-cases or controls only. Self-reported exposure to organophosphate (OP) nerve gas was a strong predictor for lower MDA level in veterans with GWI.

**Conclusion:**

Past pesticide exposures predicted lower MDA in both veterans with GWI and in healthy controls.

**Supplementary Information:**

The online version contains supplementary material available at 10.1186/s40779-021-00337-0.

## Background

Malondialdehyde (MDA) is a measure of peroxidized lipids, and has been considered as a good candidate general marker of oxidative stress (OS) by an NIH working group based on assessments involving carbon tetrachloride (CCl_4_): “It is concluded that measurements of MDA … in plasma and urine … are potential candidates for general biomarkers of oxidative stress” [1]. There is a need for information on how robust and generalizable MDA could serve as the marker of OS, both current and past, in different settings and within groups with other signatures indicative of OS injury.

Ground operation in the 1990–1991 Persian Gulf War lasted only 4 days, and many personnel was not involved in actual combat, but approximately 1/3 of the 700,000 US personnel deployed in the war later developed a variety of chronic health problems, now collectively termed “Gulf War illness (GWI)” [2, 3]. Causes of GWI are complex, but involves a range of new and unique environmental exposures, including oil fires, depleted uranium bombs, anthrax vaccine, the highest maximal number of multiple vaccines, and most notably acetylcholinesterase inhibiting agents, such as organophosphate (OP) and carbamate subclasses [4] and pesticides [5–8].

Many of the above-mentioned exposures cause OS [9]. OS is also implicated in some component symptoms of GWI [9]. In addition, mitochondrial impairment has been documented in veterans with GWI [10, 11]. The relationship between MDA and impaired mitochondria is controversial. Some evidence suggests that mitochondrial impairment leads to more free radicals in the process of energy production [12], and thus increased MDA. Other studies, however, supported reduced MDA with mitochondrial impairment. (There is precedent for association between some exposures to increased OS in many studies [13, 14], but decreased MDA under other circumstances [15].) Since membrane arachidonic acid relates positively to mitochondrial function [16, 17], and since impaired mitochondrial function has been reported in veterans with GWI [10, 11], membrane arachidonic acid might be depressed in veterans with GWI. (Arachidonic acid has been reported to inhibit mitochondrial function—so the relationship between lowered arachidonic acid and impaired mitochondrial function might reflect decreased arachidonic acid [18].) Moreover, reduced fatty acid beta-oxidation caused by bioenergetic impairment in GWI can lead to reduced oxidation of arachidonic acid and its products [19].

In the current study, we compared blood MDA concentration in veterans with GWI versus healthy controls, and examined the potential association between MDA with a variety of exposures.

## Methods

This observational study in community-dwelling individuals in Southern California sought to compare blood malondialdehyde in cases versus controls, and to assess exposure relations to MDA. The study was approved by the UCSD Human Research Protections Program (HRPP). All subjects gave written informed consent to participate.

### Design, setting and participants

This study was observational. A total of 81 subjects, including 41 veterans with GWI and 40 healthy controls, were recruited from October 2011 to May 2014. Among these subjects, 40 veterans with GWI and 40 healthy controls were matched in sex, age (within 4 years) and ethnicity (half match). Participants were community-dwelling individuals primarily from Southern California.

*Cases* To qualify as having GWI, subjects must have been deployed to the Gulf theater at any point between August 1990 and July 1991, and meet the Centers for Disease Control and Prevention (CDC) and Kansas inclusion criteria for GWI [20, 21]. CDC criteria require symptoms for at least 6 months arising since the Gulf War in at least two of three domains of fatigue/sleep, mood-cognitive, and musculoskeletal [20]. The Kansas criteria require symptoms for at least 6 months arising during or after Gulf deployment in at least 3 of 6 domains of fatigue/sleep, pain, neurological/cognitive/mood, respiratory, gastrointestinal, and dermatologic [21]. Symptoms in an individual domain must be at least moderate in severity and/or must be multiple [21]. The unmatched, 41st case was excluded from case–control comparisons, but included in analyses that predict MDA by exposures.

*Controls* Controls must be nonveterans, and meet neither Kansas nor CDC symptom inclusion criteria for GWI. Controls were also required not to meet Kansas exclusion criteria (i.e., not having other health conditions that could be confused for GWI, such as lupus or multiple sclerosis).

### Test-sets and sample size

MDA was compared in two sets of 20 pairs each (“**test-sets**”), with the unmatched 41st case in the second test-set. The first test-set was conducted during the period of participant visits, and the second test-set following the conclusion of participant visits and sample collection. For paired testing, this sample provides 94% power to detect a 0.40 standard deviation effect size, with a two-sided alpha of 0.05 (G*Power, version 3.1.9.7, HHU, Düsseldorf, Germany—see Additional file 1 for citation information). For unpaired testing, the sample provides 80% power to detect 0.63 standard deviation effect size.

### Exposure assessment

Participants completed surveys regarding environmental exposures in and out of the Gulf. Because of difficulties quantifying many types of exposure, self-rating was qualitative, rated as no (0), unsure (0.5), or yes (1).

### MDA assessment

Plasma MDA concentration was determined using the MDA-586 method from Oxis International, based on the reaction of the chromogenic N-methyl-2-phenylindole (NMPI) with MDA at 45 °C. One molecule of MDA reacts with 2 molecules of NMP to yield a stable carbocyanine dye. Absorbance was detected at 586 nm. Samples were archived at − 80 °C until analysis. Analysis of samples was conducted in two test-sets, as described above. Samples were shipped to the analysis site on dry ice with overnight shipping.

### MDA_1_

To account for possible effects of archive time, a regression predicting MDA by time-to-test (i.e. archive time) and ln(time-to-test) was performed using controls only. For both cases and controls, MDA_1_ was calculated as the departure of measured MDA from predicted MDA by this regression. We calculated the departure of measured MDA from that predicted by time-to-test variables—the portion of the variation in MDA not attributable to archive time.

### Composite variable creation

Because of high collinearity among pesticide-herbicide and fuel-solvent exposures, with pesticide exposures showing the larger coefficients in MDA prediction, a composite pesticide variable was created. A score of 1 was assigned if exposure to any of OP, organochlorine, or lice treatment is present; a score of zero was assigned otherwise.

### Statistical analysis

Continuous variables were compared using Student’s *t*-test. Categorical variables were compared using the chi-squared test. There were no missing data on the outcome variable of MDA. MDA was compared in cases versus controls by Student’s *t*-test (MDA_1_) and regression with robust standard errors, to assess prediction of MDA_1_ by case status. Prediction of MDA (by exposures) employed regression analysis with robust (heteroskedasticity-independent) standard errors, excluding those who rated an exposure as “unsure.” The analysis was conducted in the total sample, in cases, and in controls separately, with adjustment for the time variables. To address potential outcome bias that could arise from disparities in archive time, findings were adjusted for archive time and ln (archive time). In these regressions adjusted for time variables, MDA rather than MDA_1_ was used. The relative consistency of key findings was assessed separately for first versus second test-sets, and for cases and controls. The final exposure regressions in the total sample were adjusted for case status and test-set; and replicated in each test-set adjusted for case status. For analyses involving exposures that were rated as unsure, or (rarely) were unrated, the participant was dropped from the analysis except as stated. Missing data were not imputed, but no participants were missing MDA values, the composite pesticide value, or archive time values. Thus, none were missing the main outcome, exposure predictor, and/or covariate values employed in this paper. Stata® (versions 8.0 and 12.0, College Station, Texas, USA) was used. The 2-sided *P* values < 0.05 designated statistical significance.

## Results

### Participant characteristics

Table 1 shows demographic characteristics for the 80 paired participants, and by case and control status, and in the first and second sets of 20 pairs. Characteristics of the 81st (unpaired) participant are in the table legend. Data for the full set (and broken down by case–control status) for the full 81 are provided in Additional file 1: Table S1 (see Additional file 1). The first set of 20 case–control pairs included more Caucasians and was more ethnically homogeneous (*P* = 0.013). Cases were more likely to be married than controls.

**Table 1:** Participant characteristics: demographics for matched set of cases and controls

Characteristics	Combined				1st test-set			2nd test-set			*P* value		
	All (*n* = 80)	Case (*n* = 40)	Ctrl (*n* = 40)	*P*	Case (*n* = 20)	Ctrl (*n* = 20)	*P*	Case (*n* = 20)	Ctrl (*n* = 20)	*P*	All	Case	Ctrl
Age (years, mean ± *SD*)	50 ± 7.3	50 ± 7.1	49 ± 7.5	0.88	51 ± 6.9	51 ± 7.6	0.81	48 ± 7.2	48 ± 7.5	0.98	0.14	0.23	0.38
Male (%)	93	93	93	1.0	90	90	1.0	95	95	1.0	0.40	0.55	0.55
Married (%)	54	68	40	0.014	65	35	0.058	70	45	0.11	0.50	0.74	0.52
Ethnicity (%)^a^													
Caucasian	55	55	55	1.0	70	70	1.0	40	40	1.0	0.007	0.057	0.057
Hispanic	15	15	15	1.0	0	0	1.0	30	30	1.0	< 0.001	0.008	0.008
African American	20	20	20	1.0	25	25	1.0	15	15	1.0	0.26	0.43	0.43
Asian	7.5	7.5	7.5	1.0	0	0	1.0	15	15	1.0	0.011	0.072	0.072
Native American	2.5	2.5	2.5	1.0	5.0	5.0	1.0	0.0	0.0	1.0	0.15	0.31	0.31

The unmatched 81st participant (case) was an unmarried, African American male in his 60 s. *P* values are from *t*-tests of difference in mean (continuous variables) and chi-squared tests (categorical variables)

*Ctrl* control, *SD* standard deviation, *MDA* malondialdehyde

^a^ Designations for specific ethnicities are based on the matched half, where half-matches were used. The first-set (batch) evaluated MDA in the first 20 case–control pairs. The second-set (batch) evaluated the remaining participants

Symptoms, as classified by the Kansan criteria, in the cases versus control are shown in Table 2. The military characteristics of the cases are shown in Table 3.

**Table 2:** Participant characteristics: Kansas criteria qualifying symptom domains (mean ± *SD*)

Characteristics	1st test-set			2nd test-set			Combined test-sets			*P* value 1st versus 2nd set comparison		
	Case (*n* = 20)	Ctrl (*n* = 20)	*P*	Case (*n* = 20)	Ctrl (*n* = 20)	*P*	Case (*n* = 20)	Ctrl (*n* = 20)	*P*	All	Case	Ctrl
Fatigue-sleep	7.7 ± 2.4	0.30 ± 0.66	< 0.0001	7.2 ± 3.9	0.05 ± 0.22	< 0.0001	7.4 ± 3.2	0.18 ± 0.50	< 0.0001	0.38	0.63	0.12
Pain	6.1 ± 2.2	0.10 ± 0.31	< 0.0001	4.8 ± 2.4	0.15 ± 0.4	< 0.0001	5.4 ± 2.4	0.13 ± 0.40	< 0.0001	0.38	0.082	0.70
Neurological	19.0 ± 9.2	0.30 ± 0.66	< 0.0001	18.0 ± 7.6	0.05 ± 0.22	< 0.0001	18.0 ± 8.4	0.18 ± 0.50	< 0.0001	0.82	0.75	0.12
Skin	2.3 ± 0.47	0.05 ± 0.22	< 0.0001	1.4 ± 1.8	0 ± 0	0.0014	1.9 ± 2.0	0.025 ± 0.16	< 0.0001	0.21	0.15	0.32
Gastrointestinal	3.6 ± 2.4	0.05 ± 0.22	< 0.0001	3.8 ± 2.3	0 ± 0	< 0.0001	3.7 ± 2.3	0.025 ± 0.16	< 0.0001	0.86	0.74	0.32
Respiratory	2.2 ± 1.9	0.05 ± 0.22	< 0.0001	2.3 ± 2.2	0 ± 0	< 0.0001	2.3 ± 2.0	0.025 ± 0.16	< 0.0001	0.95	0.88	0.32
#Symptoms Rated 2 +	5.1 ± 0.97	0.20 ± 0.41	< 0.0001	4.7 ± 1.0	0.05 ± 0.22	< 0.0001	4.9 ± 1.0	0.13 ± 0.33	< 0.0001	0.63	0.21	0.16

The first-set (batch) evaluated MDA in the first 20 case–control pairs. The second-set (batch) evaluated the remaining participants. Values for the fatigue-sleep, pain, neurological, skin, gastrointestinal, and respiratory domains reflect the average sum of individual participants’ ratings (not present, mild, moderate, or severe) of the symptoms in each domain. Ratings for each symptom were given one of the following values: 0 = not present or onset prior to Gulf War; 1 = mild; 2 = moderate; 3 = severe. The sum range was 0–12 for the fatigue-sleep domain; 0–9 for the pain domain; 0–42 for the neurological domain; 0–6 for the skin domain; 0–9 for the gastrointestinal domain; and 0–9 for the respiratory domain. The “# Symptoms Rated 2 + ” category is defined as the number of domains having a score of 2 or greater. The range of values is 0–6. *P* for difference values were calculated by performing t-tests

*Ctrl* control, *SD* standard deviation, *MDA* malondialdehyde

**Table 3:** Military characteristics: Gulf War veteran subsample

Characteristics	Case vs control comparisons (*n*)			*P* value 1st versus 2nd test-set comparison
	All (*n* = 40)	1st test-set (*n* = 20)	2nd test-set (*n* = 20)	
Rank				0.035
Enlisted	36	16	20	
Officer	4	4	0	
Branch				0.25
Army	12	5	7	
Marine corps	12	4	8	
Navy	11	8	3	
Air force	5	3	2	
Status				
Regular	35	18	17	0.83 (NS)
Reserves	3	1	2	
National guard	2	1	1	

*P* values were based on Chi-2 testing. The first-set (batch) evaluated MDA in the first 20 case–control pairs. The second-set (batch) evaluated the remaining participants

*MDA* malondialdehyde

### MDA comparison

MDA_1_ was significantly lower in cases than controls in the full sample, as well as in both first and second sets (Table 4). MDA_1_ in the control was lower in the first than the second set. In regression with robust *SE*, lower MDA_1_ was predicted by case status: case beta = −5.7 (*SE* = 1.80), *P* = 0.002.

**Table 4:** MDA_1_ difference in case versus control

	All (*n* = 80)	1st test-set (*n* = 40)	2nd test-set (*n* = 40)	*P* value 1st versus 2nd test-set
MDA_1_ (µmol/L, mean ± *SD*)^a^				
Combined case + Control	−2.9 ± 8.5	−4.6 ± 6.3	−1.1 ± 10.0	0.064
Cases	−5.7 ± 8.4	−6.4 ± 6.1	−5.1 ± 10.0	0.63
Controls	4.9e−06 ± 7.7	−2.9 ± 6.1	2.9 ± 8.2	0.016
*P* value	0.0021	0.076	0.011	–

Case minus control values. Values are negative because MDA was lower in cases than in controls

^a^MDA_1_ is the departure of MDA from values predicted by time-to-test variables and constant—that is, MDA_1_ captures the variation in MDA that cannot be ascribed to time-to-test. In regression with robust *SE*, MDA_1_ was significantly negatively predicted by case status: case beta = −5.7 (*SE* = 1.80), *P* = 0.002. The first-set (batch) evaluated MDA in the first 20 case–control pairs. The second-set (batch) evaluated the remaining participants

*SD* standard deviation, *MDA* malondialdehyde, *MDA*_*1*_ departure of measured MDA from predicted MDA, – No data

### Exposures

Additional file 1: Table S2 shows each self-rated exposure (#no-#yes), in the total sample (“all”), in cases and controls, and in the first and second test-sets (see Additional file 1). Exposure rated as “uncertain” was excluded from the data. The difference between summed yes and no values and 41 (for cases) or 40 (for controls) provides the number that rated the exposure as unsure or (rarely), did not rate the exposure. These are presented as numbers rather than percentages. Exposures include those of potential relevance for the Gulf. Additional file 1: Table S3 shows self-ratings for Gulf-specific exposures (assessed only in Gulf War veterans) (see Additional file 1).

Military-specific exposures were generally asked only of cases (shown in Additional file 1: Table S3). However, we inquired about the history of Botulinum toxoid vaccine in both cases and controls. This vaccine was used in a minority of those who were deployed (though its use has been linked to GWI [22], so participants with GWI, among deployed veterans, may be disproportionately exposed). Consistent with this, only four among the 40 veterans, and none among the 40 controls, reported this exposure—numbers that reassure against widespread overreporting, overall or among affected veterans.

Of note, the first set of cases (and to a lesser extent, controls) had higher fractions exposed to a number of pesticide-related exposures, as well as fuel-solvent and vaccine exposures, than the second set. For example, among controls, there were 8 exposures with the exposed number greater by at least three in the first group, versus one exposure greater by at least 3 in the second.

### Relation of MDA to exposures

Additional file 1: Table S4 shows the prediction of MDA, by candidate individual MDA predictors, after adjustment for archive time (see Additional file 1). Exposures are included if they showed a significant relation to MDA in the full sample. (For the larger set of assessed exposures, see Additional file 1: Table S2.) Two main categories of exposures dominate this table: fuel-solvent-related and pesticide-herbicide-related. Second-generation antipsychotics also appear significant in cases, but this is based on a single case for each of the antipsychotic agents. The herbicide treatment at work variable is also based on a few. There is an additional representation of several vaccines, in cases only.

Several additional fuel-solvent and pesticide variables were negative and significant in at least one subcategory (particularly controls) though not in the total sample, and did not meet criteria for inclusion.

Organochlorines and lice treatment were consistently related to lower MDA, with *P* ≤ 0.001 in three categories including the total sample, adjusted for case status. Herbicide treatment at the workplace was also related to lower MDA, but the small number that reported the exposure limited the confidence of the finding.

Lindane, an organochlorine, is the primary lice treatment used. Lice treatment was asked separately because it may have been known for its function, rather than by its pesticide class. So, the observed associations may particularly support a powerful negative association of past organochlorine pesticide exposure to MDA.

Additional file 1: Table S5 shows univariable MDA prediction by Gulf-specific exposures, with and without adjustment for the test-set (see Additional file 1). The exposures that predicted MDA with *P* < 0.1 on both regressions are shown. Since test-set differences may arise from differences in relevant exposures (more exposures in the first group), the test-set adjusted version is not necessarily more valid. All regression analyses adjust for time-to-test and ln (time-to-test). Only exposures with an apparent relation to MDA are shown. Other than the positive relationship for “saw smoke,” negative relationships predominate, related to potential nerve gas attack [or other exposures that may have led the chemical alarms to sound, gas masks or nuclear biological chemical (NBC) protection suits to be donned].

The pesticide exposures are strongly intercorrelated. Fuel and solvent exposures are also strongly correlated to one another and correlated to pesticide exposures.

Table 5 shows the prediction of MDA by the composite pesticide variable. The pesticide variable was significant in all groups—cases, controls, first test-set, second test-set. The fraction exposed to OPs, organochlorines, and lice treatment was ~ 10, 5, and 2.5 times greater, respectively, in the first relative to the second group (25%, 12.5%, and 12.5% in the first set, vs. 9.6%, 4.8%, and 2.4% in the second, respectively). The difference between fractions exposed in the first and second test-set was significant: e.g. *P* = 0.009 for the presence of at least one such exposure, and *P* = 0.002 for OPs specifically. All regressions were performed with adjustment for time-to-test and ln(time-to-test), unless otherwise stated. Time-to-test variables were significant for controls, and for the total sample adjusted for case status; but did not alter coefficients or significance of the pesticide (composite) variable in any analysis, and did not approach significance for other analyses.

**Table 5:** Prediction of MDA by pesticides (composite variable)

MDA	*β* (*SE*)	*P*
Adjusted for case status and 1st versus 2nd set (*n* = 81)	− 5.3 (1.3)	< 0.001
In case and in control separately adjusted for 1st versus 2nd set		
Case (*n* = 41)	− 5.2 (1.7)	0.004
Control (*n* = 40)	− 8.0 (1.7)	< 0.001
In first and in second set separately adjusted for case status		
1st set (*n* = 40)	− 4.5 (1.3)	0.001
2nd set (*n* = 41)	− 7.0 (3.0)	0.026

Regressions with robust standard errors, adjusted for time-to-test and ln(time-to-test). For the composite pesticide variable, exposure was deemed “present” if there was exposure to any of the components; otherwise it was coded as absent. Exposure was present for 11 cases, 6 controls, 13 in the first test-set, and 4 in the second. For the remainder, exposure was absent. The first-set (batch) evaluated MDA in the first 20 case–control pairs. The second-set (batch) evaluated the remaining participants

*SE* standard error, *MDA* malondialdehyde

Veterans were also asked about Gulf-specific exposures, and one of these is of potential relevance, given the pesticide finding incorporating OPs. A regression model for cases, assessing variables that cannot be replicated in controls, prioritized exposures tied to potential nerve agent exposure (OP). Inclusion of these variables led to an R^2^ of 0.60, founded on large negative associations of potential OP (nerve agent) exposure, and positive associations of a composite measure of (history of use of) second generation antipsychotics (2GA) (this includes the three 2nd generation antipsychotics, olanzapine, risperidone, quetiapine [23])—to which, however, few were exposed.

In the two-variable model (with full adjustments for time-to-test, ln(time-to-test)), test-set—but without the antipsychotic variable), coefficients are identical and *P* values significant with or without exclusion of those who cite their exposure as “unsure”—but the sample size is reduced to 25 if those citing exposure as “unsure” are excluded. To better support the larger number of adjustment variables, the models showed include those who rated the variable as “unsure.” If those rating an exposure as “unsure” in the 3-variable model are excluded, despite the smaller sample size, each predictor remains significant.

Models separately optimized for cases and controls are shown in Additional file 1: Tables S6 and S7, respectively (see Additional file 1). These do not have the authority conferred by replication of findings across cases and controls. The optimized case model includes chemical attacks and composite pesticides as negative predictors.

## Discussion

The current study showed lower plasma MDA concentration in veterans with GWI than in matched healthy controls. Low MDA was predicted by past pesticide exposure (a finding that was separately affirmed in cases and in controls).

MDA is viewed as a biomarker of oxidative stress based on a CCl_4_ exposure model; and oxidative stress is thought to be a feature of GWI, with findings extending to animal models of GWI (see Additional file 1). Many arachidonic acid products appear to be depressed in veterans with GWI. A preliminary muscle biopsy study [24] supports impaired mitochondrial fatty acid oxidation in GWI, linking MDA with impaired mitochondrial function in this setting.

MDA was depressed in veterans with GWI in both the first and second test-sets in the current study. The association between depressed MDA with past exposures to some classes of pesticides was evident both in veterans with GWI and healthy controls. We underscore that pesticides were used in the Gulf to protect against insect vectors of disease (and indeed, rates of infectious diseases were low during the Gulf relative to many prior conflicts). Qualitative concordance of key findings in cases and controls strengthens confidence in the findings.

In GWI cases, proxies for exposure to OP nerve agent—to which exposure occurred during the Khamisiyah munitions depot demolition and other episodes [25]—predicted lower MDA, that swamped pesticide exposure as a contributory predictor of lower MDA. This, coupled with the larger absolute magnitude of the coefficient for OP exposure (relative to other pesticide exposures) in controls, suggest the possibility that MDA depression may particularly be associated with (past) OP exposure. A model in GWI cases that considered only putative nerve agent related exposure and second-generation antipsychotic exposure (which predict in the opposite direction) produced an R^2^ of 0.60—that is, it appeared to explain a high fraction of the variance. The antipsychotic findings were based on low numbers—but more than one agent in the class contributed.

In controls, there is less potential for competing and unmeasured exposures to drive spurious exposure-marker relationships. In this group, it is less likely a given exposure serves as a proxy for higher exposure to many unrelated agents, as might be hypothesized for the veteran group. Additionally, there are expected to be fewer other exposures to add variance; and fewer who cited uncertainty about exposures. Unlike the situation for many veterans during the Gulf conflict, controls may often have been in charge of their exposure. This adds relative confidence to findings in controls, in whom each of several pesticide-herbicide agents was independently associated with lower MDA in a multivariable regression model. Greater exposures in the first test-set may explain the apparently lower MDA in control subjects in the first versus second group.

Multiple toxic co-exposure classes in veterans with GWI were correlated with one another, which may add uncertainty to the foundations of exposure relations in Gulf War veterans. Such uncertainty could be attenuated by replication in controls.

A small number of veterans were exposed to second-generation antipsychotic drugs—olanzapine, risperidone or quetiapine. These agents are widely used for many non-psychotic conditions. Gulf War veterans may have particular prospects for exposure, both due to more frequent contact with the healthcare system and because treatment guidelines put forth by the Department of Veterans Affairs (VA) for GWI, subsumed under the rubric of chronic multisymptom illness, have endorsed—without supportive evidence—psychiatric medications for this condition [26]. Though our analyses involving these exposures were based on small numbers, providing attendant uncertainty to estimates, the large magnitude of effect (positive prediction of MDA), and consistency across each of the agents (significant whether assessed separately or grouped together), adds modest confidence to the finding. This is consistent with previous reports that these agents are oxidative stressors [27].

Exposures may alter lipids, not just peroxidation of them; and depression or elevation of the lipid can lead to depression or elevation of its oxidized version. This cannot be excluded as a possible factor in the prediction of higher MDA by these agents, and may explain the prediction of lower MDA by pesticides—although actual reductions in OS are an alternate possibility.

Lower MDA levels in veterans with GWI might also reflect altered lipid profiles. Lower MDA and higher 8-hydroxy-2’-deoxyguanosine (8-OHDG) have been shown to predict case status, suggesting these candidate general markers of OS show a dissociation in this setting [28]. MDA derives from arachidonic acid metabolism [29, 30]. We previously reported widespread depressions in prostaglandins and leukotrienes in veterans with GWI [31]; these are also (eicosanoid) products of arachidonic acid [32]. And in a metabolomic assessment, we found depressions in yet other arachidonate-derived products, such as 12-hydroxyeicosatetraenoic acid (12-HETE) and endocannabinoids [33]. Pesticides, as well as fuels and solvents [34], have been reported to alter lipid profiles of membranes. This supports prospects for alterations in lipid profiles in the blood, because membrane phospholipids serve as reservoirs for arachidonic acid and its eicosanoid products [35].

Prior evidence supports lipidomic changes in animal models of GWI that involve exposure to pesticides [36]. Pesticides (and herbicides) are typically delivered in solvents, which may (among other ingredients [37]) influence their effects. Prior evidence supports membrane lipid changes following exposure to solvents, including increases in linoleic acid derivatives in membranes [34], as well as membrane changes and fatty acid metabolism disturbances following pesticide exposure [38].

Lipid changes could serve “adaptive” functions, for instance, in response to acute effects of OS on membrane fluidity or permeability [39], or conversely, as part of protecting against potential arachidonic acid-induced increases in OS and mitochondrial impairment [18]. Reduced arachidonic acid and products could also contribute to health issues, or both (or in principle, neither). For instance, reduced arachidonate in membranes may be tied to membrane fluidity and thereby possibly reduce adenosine triphosphate (ATP) leakage at least in some cell types [39], but this may be tied to less ATP production (albeit conceivably as an adaptive consequence) [16, 17]. Factors tied to greater membrane rigidity are also tied to greater resistance against cytotoxic stressors, so conversely this fluidizing effect may impair resistance [40]. Indeed, it could be speculated that this is a factor in elevated multiple chemical sensitivity (and heightened sensitivity to radiation in a subset of that group—radiation has also been reported to lead to membrane fluidization [15]) in veterans with pesticide exposure [41]. The possibility that effects are adaptive must be considered in the context of the evolutionarily recent and primarily human introduction of the relevant chemicals. Irrespective of these considerations, whether low MDA meaningfully reflects lower OS in this setting, assessed by non-lipid measures, requires further study. There is also the possibility of “oxidative preconditioning” in which prior exposure to an oxidative stressor leads to upregulation of antioxidant defenses which could produce true reductions in oxidative stress [42, 43], as the basis for depressed MDA. Finally, veterans with GWI might be taking more antioxidants. However, the lack of depression of other oxidative stress markers (not shown), including total antioxidant capacity (and see prior discussion of 8-OHDG), and presence of depressions of other arachidonic acid derivatives, may render this less likely.

Lipid alterations from OPs, and membrane effects deriving from these, impair mitochondrial ATP production [44]. These might contribute to or underlie the bioenergetic deficits that have been reported in veterans with GWI [10, 11]. Membrane arachidonic acid relates positively to mitochondrial function [16, 17]. Thus, the mitochondrial impairment in GWI may portend lower membrane arachidonic acid reservoirs; and moreover, reduced fatty acid beta-oxidation, from bioenergetic impairment in GWI, may reduce the oxidative modification of arachidonic acid [19].

A strength of the current study is the use of the more sensitive Kansas symptom criteria for GWI, as well as the CDC symptom criteria. Also, cases and controls were matched for age, sex and ethnicity. Because veterans were exposed to many agents, providing concern that exposures may serve as proxies for correlated exposures, assessment of exposure relations to MDA in a control group, including some who had exposure to candidate agents, is a strength—and provides important corroboration of key findings.

A key limitation in the current study lies in the fact that exposures were based on recall, and thus subjected to significant recall bias. Also, recruitment was not contingent on the presence or absence of specific exposures. Some controls might be biochemically more similar to cases, if they have had similar exposures. However, the ability to assess exposure relations separately in controls relies on the presence of exposures in some controls. (The second set of participants—including the latter 20 case–control pairs, in whom MDA was assessed separately—included few who cited exposure to assessed pesticides, reducing the ability to assess relations of these exposures to MDA in the second set.) Controls did not have Gulf-specific exposures: analyses here focus on exposures that could be relatively corroborated in controls. Exposures in Gulf War veterans, particularly, may be correlated with other exposures not included in the current study. However, concordance of key findings in controls, where this concern is markedly attenuated, bolsters confidence in key findings. Additionally, among Gulf-specific exposures, ties to MDA were predominantly seen for exposures that are expected to correlate with nerve agent (OP) exposure. The direction of association to MDA was the same for OP pesticide exposures, in cases and controls, providing relative corroboration. Quantitation of the exposure, for many of these exposures of interest, can be difficult.

The study assessed only plasma MDA; tissue MDA (e.g. brain tissue) may not be similarly depressed. Assessment of MDA was conducted well after Gulf exposures. This is a potential strength as well as a weakness, since there is particular cause to look at those markers and mechanisms for which alterations are present or sustained well after exposure in veterans with GWI, in whom health issues have been sustained; and to understand long term health effects of exposures. Increases in OS have been reported in the short term, following exposure to both pesticides [45] and solvents [46, 47]. However, a number of findings underscore that long-term effects (delayed) of OPs and carbamates may differ from short-term effects [48, 49].

It is possible that in long-term follow-up after pesticide exposure, lower MDA could arise from actually lower extracellular OS (OS is compartmentalized [50]; mitochondrial OS, for instance, may remain higher). Such an effect could occur from sustained adaptive upregulation of antioxidant defenses, or reduced fatty acid beta-oxidation [19], or conceivably intake of antioxidant supplements. However, the latter would be less able to explain an association of pesticide exposure to lower MDA in healthy controls.

## Conclusions

Past pesticide and OP exposure are associated with long-term depression of MDA, and likely other arachidonic acid products. The finding raises questions about whether MDA and markers of lipid peroxidation are valid markers of OS. Concurrent assessments of markers that do not rely on lipids, such as 8-OHDG, a marker of oxidative injury to deoxyribonucleic acid (DNA); or ratio of oxidized to reduced quantities like glutathione or coenzyme Q10, may be necessary. Attention should also be given to the relation of time after exposure to effect on candidate markers of OS.

## Supplementary Information

**Additional file 1**. Additional files The following are available as supplementary data in the Additional files: **Table S1** Demographics of study participants (*n* = 81). **Table S2** Exposures in cases and controls. **Table S3** Gulf-specific exposures in first vs second test-set. **Table S4** Exposure predictors of MDA – exposures significant on univariable analysis in the total sample. **Table S5** Gulf-specific exposure predictors of MDA – exposures significant on univariable analysis variables assessed for cases only (*n* = 41). **Table S6** Optimized MDA Model for Cases (*n* = 41). **Table S7** MDA – multivariable prediction in controls.

## Data Availability

Adequately deidentified data used and/or analyzed during the current study are available from the corresponding author on reasonable request.

## Abbreviations

12-HETE: 12-Hydroxyeicosatetraenoic acid
2GA: Second generation antipsychotic
8-OHDG: 8-Hydroxy-2′-deoxyguanosine
ATP: Adenosine triphosphate
CCl_4_: Carbon tetrachloride
CDC: Centers for disease control and prevention
Ctrl: Control
DNA: Deoxyribonucleic acid
GWI: Gulf War illness
MDA: Malondialdehyde
MDA_1_: Departure of measured MDA from predicted MDA
NBC: Nuclear biological chemical
NMPI: N-methyl-2-phenylindole
OP: Organophosphate
OS: Oxidative stress
SD: Standard deviation
SE: Standard error
VA: Department of Veterans affairs
