# Supplementary material for: Lower blood malondialdehyde is associated with past pesticide exposure: findings in Gulf War illness and healthy controls
Source: Mil Med Res. 2021 Aug 17;8:46. doi: 10.1186/s40779-021-00337-0 (PMC8369730; doi:10.1186/s40779-021-00337-0)
Supplement: Supplementary file 1 — Additional file 1. Additional files The following are available as supplementary data in the Additional files: Table S1 Demographics of study participants (n = 81). Table S2 Exposures in cases and controls. Table S3 Gulf-specific exposures in first vs second test-set. Table S4 Exposure predictors of MDA – exposures significant on univariable analysis in the total sample. Table S5 Gulf-specific exposure predictors of MDA – exposures significant on univariable analysis variables assessed for cases only (n = 41). Table S6 Optimized MDA Model for Cases (n = 41). Table S7 MDA – multivariable prediction in controls. [file 40779_2021_337_MOESM1_ESM.docx]

**SUPPLEMENTAL MATERIAL**

**Lower blood malondialdehyde is associated with past pesticide exposure: findings in Gulf War illness and healthy controls**

Beatrice Alexandra Golomb, Sridevi Devaraj, Alexis K. Messner, Hayley Jean Koslik, Jun Hee Han and Barnabas Yik

Power calculations made use of G*Power 3.1.9.7 [1, 2].

**Table S1** Demographics of study participants (*n =* 81)

| Demographic features | All  (*n =* 81) | Cases  (*n* = 41) | Controls  (*n* = 40) | *P value*  for difference  (case vs control) |
| --- | --- | --- | --- | --- |
| Age (years, mean±*SD*) | 49.8 ± 7.5 | 50.1 ± 7.6 | 49.5 ± 7.5 | 0.68 |
| Male (%) | 92.6 | 92.7 | 92.5 | 0.98 |
| Ethnicity |  |  |  |  |
| Caucasian | 54.3 | 53.7 | 55.0 | 0.90 |
| African American (%) | 21.0 | 22.0 | 20.0 | 0.83 |
| Latino | 14.8 | 14.6 | 15.0 | 0.96 |
| Asian | 7.4 | 7.3 | 7.5 | 0.98 |
| Native American | 2.4 | 2.4 | 2.5 | 0.99 |
| Married (%) | 51.8 | 65.9 | 38.1 | 0.011 |

*SD* standard deviation.

**Table S2** Exposures in cases and controls

| Exposure | All  *n* = 80 | Both sets  *n* = 80 | | | 1st test-set  *n* = 40 | | | 2nd test-set  *n* = 40 | | | 1st vs 2nd test-set  *P value* | | |
| --- | --- | --- | --- | --- | --- | --- | --- | --- | --- | --- | --- | --- | --- |
|  |  | Ctrl | Case | *P*  (Chi2) | Ctrl | Case | *P* | Ctrl | Case | *P* | All | Ctrl | Case |
| Burning fuels (e.g., combustion products, coal, wood, oil) | 39-38 | 33-7 | 6-31 | <0.001 | 16-4 | 4-15 | <0.001 | 17-3 | 2-16 | <0.001 | 0.91 | 0.68 | 0.41 |
| Carbon monoxide (e.g. car malfunction/exhaust vented in; heater malfunction in home) | 60-14 | 36-4 | 24-10 | 0.034 | 18-2 | 14-5 | 0.18 | 18-2 | 10-5 | 0.088 | 0.82 | 1.0 | 0.66 |
| Diesel or petrochemical  fuel on skin | 53-23 | 36-4 | 17-19 | <0.001 | 18-2 | 7-12 | 0.001 | 18-2 | 10-7 | 0.028 | 0.27 | 1.0 | 0.19 |
| Diesel or petrochemical  fumes (e.g., exhaust) | 48-30 | 36-4 | 12-26 | <0.001 | 18-2 | 7-13 | <0.001 | 18-2 | 5-13 | <0.001 | 0.86 | 1.0 | 0.63 |
| Jet fuel | 50-28 | 36-3 | 14-25 | <0.001 | 17-2 | 6-14 | <0.001 | 19-1 | 8-11 | <0.001 | 0.35 | 0.52 | 0.43 |
| Kerosene (e.g. paraffin) | 62-15 | 39-1 | 23-14 | <0.001 | 19-1 | 11-8 | 0.006 | 20-0 | 12-6 | 0.005 | 0.42 | 0.31 | 0.58 |
| Stored fuels | 64-12 | 38-2 | 26-10 | 0.007 | 18-2 | 14-6 | 0.11 | 20-0 | 12-4 | 0.018 | 0.29 | 0.15 | 0.74 |
| Petroleum products (e.g.,  oil) | 55-22 | 35-5 | 20-17 | 0.001 | 16-4 | 10-10 | 0.047 | 19-1 | 10-7 | 0.008 | 0.19 | 0.15 | 0.59 |
| Acetone (e.g., nail polish  remover, paint thinner) | 41-36 | 27-12 | 14-24 | 0.004 | 13-6 | 9-11 | 0.14 | 14-6 | 5-13 | 0.009 | 0.57 | 0.92 | 0.27 |
| Paint, paint strippers | 42-37 | 27-13 | 15-24 | 0.010 | 16-4 | 8-12 | 0.010 | 11-9 | 7-12 | 0.26 | 0.22 | 0.091 | 0.84 |
| Other solvents, thinning agents (paint thinner) | 39-33 | 26-11 | 13-22 | 0.005 | 14-4 | 7-11 | 0.018 | 12-7 | 6-11 | 0.095 | 0.48 | 0.33 | 0.83 |
| Solvents | 54-21 | 34-5 | 20-16 | 0.002 | 15-4 | 12-8 | 0.20 | 19-1 | 8-8 | 0.002 | 0.58 | 0.13 | 0.55 |
| Degreasing solutions | 56-22 | 33-7 | 23-15 | 0.031 | 15-5 | 13-7 | 0.49 | 18-2 | 10-8 | 0.016 | 0.72 | 0.21 | 0.55 |
| Automotive products | 52-22 | 30-9 | 22-13 | 0.19 | 15-4 | 13-6 | 0.46 | 15-5 | 9-7 | 0.24 | 0.51 | 0.77 | 0.46 |
| Black Flag (spray insecticides) | 43-33 | 29-11 | 14-22 | 0.003 | 15-5 | 6-11 | 0.015 | 14-6 | 8-11 | 0.079 | 0.98 | 0.72 | 0.68 |
| Carbamate pesticides (e.g., Temik, Carbayl, Furadan) | 57-8 | 36-1 | 21-7 | 0.007 | 17-1 | 9-6 | 0.016 | 19-0 | 12-1 | 0.22 | 0.026 | 0.30 | 0.049 |
| DEET (e.g., insect repellant) | 46-25 | 35-3 | 11-22 | <0001 | 15-3 | 4-15 | <0.001 | 20-0 | 7-7 | <0.001 | 0.013 | 0.057 | 0.081 |
| Dursban | 61-3 | 37-1 | 24-2 | 0.35 | 18-0 | 13-2 | 0.11 | 19-1 | 11-0 | 0.45 | 0.59 | 0.34 | 0.21 |
| Flea or tick medicine | 55-16 | 34-5 | 21-11 | 0.031 | 17-2 | 7-8 | 0.007 | 17-3 | 14-3 | 0.83 | 0.18 | 0.68 | 0.034 |
| Fly spray | 60-15 | 36-4 | 24-11 | 0.021 | 19-1 | 12-6 | 0.024 | 17-3 | 12-5 | 0.29 | 0.73 | 0.29 | 0.80 |
| Head lice treatment (e.g.,  Lindane) | 72-7 | 38-2 | 34-5 | 0.22 | 18-2 | 16-3 | 0.59 | 20-0 | 18-2 | 0.15 | 0.22 | 0.15 | 0.59 |
| Insect repellent (e.g., Off) | 41-37 | 31-9 | 10-28 | <0.001 | 14-6 | 5-14 | 0.006 | 17-3 | 5-14 | <0.001 | 0.50 | 0.26 | 1.0 |
| Moth repellants/moth balls (naphthalene) | 66-11 | 37-3 | 29-8 | 0.077 | 18-2 | 15-4 | 0.34 | 19-1 | 14-4 | 0.12 | 0.78 | 0.55 | 0.93 |
| Organophosphates- insecticides (e.g., parathion, malathion, chlorpyrifos, phosmet, fenitrothion, diisoprylfluorophate/DFT) | 51-11 | 34-3 | 17-8 | 0.016 | 16-3 | 5-7 | 0.014 | 18-0 | 12-1 | 0.23 | 0.003 | 0.079 | 0.007 |
| Organochlorines (e.g., chlordane, heptachlor, pentachlorophenol and DDT) | 56-6 | 35-3 | 21-3 | 0.55 | 17-2 | 9-3 | 0.29 | 18-1 | 12-0 | 0.42 | 0.086 | 0.55 | 0.064 |
| Pesticide cream or spray on your skin | 52-22 | 37-3 | 15-19 | <0.001 | 19-1 | 6-11 | <0.001 | 18-2 | 9-8 | 0.011 | 0.61 | 0.55 | 0.30 |
| Pesticides on clothing or  bedding | 52-19 | 38-2 | 14-17 | <0.001 | 19-1 | 5-10 | <0.001 | 19-1 | 9-7 | 0.005 | 0.38 | 1.0 | 0.20 |
| Pyrethroid pesticides/pest repellants (e.g. permethrin-treated  clothing, etc) | 58-11 | 38-2 | 20-9 | 0.004 | 19-1 | 8-7 | 0.004 | 19-1 | 12-2 | 0.35 | 0.11 | 1.0 | 0.060 |
| Raid (ant killer) | 45-33 | 28-12 | 17-21 | 0.024 | 13-7 | 8-10 | 0.20 | 15-5 | 9-11 | 0.053 | 0.67 | 0.49 | 0.97 |
| Saw the area in which you lived fogged or sprayed  with pesticides | 47-30 | 31-9 | 16-21 | 0.002 | 14-6 | 5-15 | 0.004 | 17-3 | 11-6 | 0.15 | 0.011 | 0.26 | 0.015 |
| Other pesticides/insecticides | 60-5 | 38-1 | 22-4 | 0.057 | 18-1 | 10-4 | 0.065 | 20-0 | 12-0 | n/a | 0.022 | 0.30 | 0.044 |
| Regular pesticide treatment at place of work | 53-20 | 33-7 | 20-13 | 0.037 | 18-2 | 10-8 | 0.016 | 15-5 | 10-5 | 0.59 | 0.83 | 0.21 | 0.52 |
| Paraquat (herbicide) | 65-2 | 39-1 | 26-1 | 0.78 | 19-1 | 12-0 | 0.43 | 20-0 | 14-1 | 0.24 | 0.95 | 0.31 | 0.36 |
| Roundup (herbicide) | 54-23 | 34-6 | 20-17 | 0.003 | 18-2 | 10-8 | 0.016 | 16-4 | 10-9 | 0.070 | 0.50 | 0.38 | 0.86 |
| Wood treatment | 64-10 | 36-4 | 28-6 | 0.34 | 17-3 | 14-3 | 0.83 | 19-1 | 14-3 | 0.22 | 0.50 | 0.29 | 1.0 |
| Other herbicides (e.g.,  Weed-B-Gone) | 59-14 | 37-3 | 22-11 | 0.005 | 19-1 | 12-5 | 0.045 | 18-2 | 10-6 | 0.049 | 0.52 | 0.55 | 0.62 |
| Regular herbicide  treatment at place of work | 67-2 | 37-1 | 30-1 | 0.88 | 20-0 | 14-1 | 0.24 | 17-1 | 16-0 | 0.34 | 0.98 | 0.29 | 0.29 |
| Napthas, other | 66-4 | 35-1 | 31-3 | 0.28 | 17-1 | 16-2 | 0.55 | 18-0 | 15-1 | 0.28 | 0.33 | 0.31 | 0.62 |
| Dry cleaned clothing | 45-33 | 29-9 | 16-24 | 0.001 | 14-5 | 7-13 | 0.015 | 15-4 | 9-11 | 0.029 | 0.49 | 0.70 | 0.52 |
| Fabric softeners, dryer sheets | 43-36 | 24-15 | 19-21 | 0.21 | 12-7 | 9-11 | 0.26 | 12-8 | 10-10 | 0.53 | 0.92 | 0.84 | 0.75 |
| Mercury (e.g. tuna, dental amalgam fillings,  thermometer, light bulbs) | 45-29 | 27-11 | 18-18 | 0.064 | 15-3 | 9-10 | 0.022 | 12-8 | 9-8 | 0.67 | 0.48 | 0.11 | 0.74 |
| Arsenic (e.g., rat poison) | 64-6 | 39-0 | 25-6 | 0.004 | 19-0 | 15-3 | 0.063 | 20-0 | 10-3 | 0.024 | 0.88 | n/a | 0.66 |
| Selenium (e.g., copiers, glass manufacturing) | 64-6 | 38-0 | 26-6 | 0.005 | 18-0 | 13-3 | 0.054 | 20-0 | 13-3 | 0.043 | 0.94 | n/a | 1.0 |
| Cadmium (e.g. batteries) | 59-13 | 36-2 | 23-11 | 0.003 | 17-1 | 11-6 | 0.028 | 19-1 | 12-5 | 0.045 | 0.68 | 0.94 | 0.71 |
| Chromium (e.g., coating on car parts) | 68-9 | 36-3 | 32-6 | 0.27 | 17-2 | 17-3 | 0.68 | 19-1 | 15-3 | 0.24 | 0.75 | 0.52 | 0.89 |
| Cobalt (e.g., batteries, pigments, paints, magnets) | 62-10 | 37-2 | 25-8 | 0.019 | 18-1 | 14-4 | 0.13 | 19-1 | 11-4 | 0.070 | 0.93 | 0.97 | 0.77 |
| Copper (e.g. electrical  machines, roofer, copper- containing fungicides) | 57-20 | 35-5 | 22-15 | 0.005 | 17-3 | 12-6 | 0.18 | 18-2 | 10-9 | 0.010 | 0.65 | 0.63 | 0.39 |
| Iron (e.g., cars, ships, machine tools) | 56-21 | 36-4 | 20-17 | <0.001 | 18-2 | 11-7 | 0.036 | 18-2 | 9-10 | 0.004 | 0.49 | 1.0 | 0.40 |
| Lead (e.g. bullets, solder,  pewter, construction) | 47-31 | 34-6 | 13-25 | <0.001 | 16-4 | 7-12 | 0.006 | 18-2 | 6-13 | <0.001 | 0.82 | 0.38 | 0.73 |
| Manganese (e.g. iron and steel production) | 68-6 | 39-0 | 29-6 | 0.007 | 19-0 | 14-2 | 0.11 | 20-0 | 15-4 | 0.030 | 0.48 | n/a | 0.50 |
| Zinc (e.g., mining, cars, construction) | 67-4 | 38-1 | 29-3 | 0.22 | 18-1 | 16-1 | 0.94 | 20-0 | 13-2 | 0.093 | 0.98 | 0.30 | 0.47 |
| Radiation treatment for cancer or other conditions | 75-3 | 38-1 | 37-2 | 0.56 | 18-1 | 18-1 | 1.0 | 20-0 | 19-1 | 0.31 | 0.53 | 0.30 | 0.97 |
| X-rays, radiation | 32-46 | 22-17 | 10-29 | 0.006 | 10-9 | 5-15 | 0.076 | 12-8 | 5-14 | 0.034 | 0.65 | 0.64 | 0.93 |
| Other radiation | 54-15 | 36-1 | 18-14 | <0.001 | 17-1 | 8-8 | 0.003 | 19-0 | 10-6 | 0.003 | 0.35 | 0.30 | 0.48 |
| Radioactive chemicals | 54-10 | 35-2 | 1-8 | 0.008 | 16-2 | 10-4 | 0.21 | 19-0 | 9-4 | 0.010 | 0.49 | 0.14 | 0.90 |
| Aerosol sprays | 38-38 | 23-14 | 15-24 | 0.039 | 12-6 | 8-12 | 0.10 | 11-8 | 7-12 | 0.19 | 0.65 | 0.58 | 0.84 |
| Air fresheners | 44-35 | 27-13 | 17-22 | 0.032 | 14-6 | 10-10 | 0.20 | 13-7 | 7-12 | 0.079 | 0.44 | 0.74 | 0.41 |
| Animal sprays | 67-5 | 38-1 | 29-4 | 0.11 | 18-1 | 15-2 | 0.48 | 20-0 | 14-2 | 0.10 | 0.64 | 0.30 | 0.95 |
| Asbestos | 51-16 | 36-1 | 15-15 | <0.001 | 17-1 | 9-7 | 0.009 | 19-0 | 6-8 | <0.001 | 0.95 | 0.30 | 0.46 |
| Mold or fungus | 52-19 | 32-7 | 20-12 | 0.064 | 16-3 | 10-8 | 0.057 | 16-4 | 104 | 0.56 | 0.56 | 0.73 | 0.36 |
| Antimalarial drugs (e.g. chloroquine, hydroxychloroquine, mefloquine-Larium) taken or prescribed | 52-18 | 38-2 | 14-16 | <0.001 | 20-0 | 6-9 | <0.001 | 18-2 | 8-7 | 0.014 | 1.0 | 0.15 | 0.46 |
| Ciprofloxacin “cipro” or other “fluoroquinolones” (levofloxacin = levoquin, ofloxacin, others ending in “floxacin”) taken or prescribed | 45-22 | 31-6 | 14-16 | 0.001 | 13-5 | 6-9 | 0.062 | 18-1 | 8-7 | 0.005 | 0.10 | 0.063 | 0.46 |
| Doxycycline taken or prescribed | 45-23 | 33-4 | 12-19 | <0.001 | 16-2 | 4-12 | <0.001 | 17-2 | 8-7 | 0.018 | 0.20 | 0.95 | 0.11 |
| Anthrax vaccine | 41-35 | 36-3 | 5-32 | <0001 | 17-2 | 1-17 | <0.001 | 19-1 | 4-15 | <0.001 | 0.37 | 0.52 | 0.17 |
| Botox vaccine | 71-3 | 39-1 | 32-2 | 0.46 | 19-1 | 14-1 | 0.83 | 20-0 | 18-1 | 0.30 | 0.49 | 0.31 | 0.86 |
| Botulinum toxoid or “BT” vaccine | 62-4 | 40-0 | 22-4 | 0.010 | 20-0 | 8-4 | 0.006 | 20-0 | 14-0 | n/a | 0.033 | n/a | 0.019 |
| Cholera vaccine | 56-13 | 38-2 | 18-11 | 0.001 | 19-1 | 7-9 | 0.001 | 19-1 | 11-2 | 0.31 | 0.047 | 1.0 | 0.024 |
| Hepatitis A vaccine | 41-24 | 24-10 | 17-14 | 0.19 | 11-7 | 6-8 | 0.31 | 13-3 | 11-6 | 0.29 | 0.10 | 0.20 | 0.22 |
| Hepatitis B vaccine | 31-36 | 18-6 | 13-20 | 0.27 | 8-10 | 5-11 | 0.43 | 10-6 | 8-9 | 0.37 | 0.18 | 0.29 | 0.35 |
| TwinRx (combined HepA and HepB) vaccine | 54-7 | 30-2 | 24-5 | 0.18 | 16-1 | 10-3 | 0.17 | 14-1 | 14-2 | 0.58 | 0.65 | 0.93 | 0.45 |
| Immunoglobulin vaccine | 46-21 | 33-2 | 13-19 | <0.001 | 17-1 | 5-12 | <0.001 | 16-1 | 8-7 | 0.008 | 0.29 | 0.97 | 0.17 |
| Meningococcal vaccine | 50-12 | 33-0 | 17-12 | <0.001 | 16-0 | 6-8 | <0.001 | 17-0 | 11-4 | 0.023 | 0.16 | n/a | 0.096 |
| Measles, mumps, rubella (MMR) vaccine | 15-55 | 9-24 | 6-31 | 0.26 | 5-12 | 3-15 | 0.37 | 4-12 | 3-16 | 0.50 | 0.77 | 0.78 | 0.94 |
| Pertussis vaccine | 41-28 | 26-12 | 15-16 | 0.092 | 13-6 | 8-8 | 0.27 | 13-6 | 7-8 | 0.20 | 0.92 | 1.0 | 0.85 |
| Plague vaccine | 54-13 | 36-1 | 18-12 | <0.001 | 17-1 | 7-10 | 0.001 | 19-0 | 11-2 | 0.077 | 0.009 | 0.30 | 0.016 |
| Poliomyelitis vaccine | 28-42 | 17-19 | 11-23 | 0.20 | 8-11 | 6-11 | 0.68 | 9-8 | 5-12 | 0.16 | 0.85 | 0.52 | 0.71 |
| Tetanus vaccine | 9-67 | 7-31 | 2-36 | 0.076 | 4-16 | 2-17 | 0.41 | 3-15 | 0-19 | 0.063 | 0.33 | 0.79 | 0.15 |
| Typhoid vaccine | 42-25 | 33-5 | 9-20 | <0.001 | 16-3 | 3-12 | <0.001 | 17-2 | 6-8 | 0.004 | 0.24 | 0.63 | 0.18 |
| Yellow fever vaccine | 44-24 | 35-2 | 9-22 | <0.001 | 16-1 | 3-14 | <0.001 | 19-1 | 6-8 | 0.001 | 0.13 | 0.91 | 0.12 |
| Number of vaccines given  (mean-SD) | 5.0  (3.6) | 3.2  (2.1) | 6.7  (3.9) | <0.000  1 | 3.6  (2.4) | 7.9  (4.6) | 0.0006 | 2.5  (1.7) | 5.5(2.8) | 0.0001 | 0.055 | 0.26 | 0.052 |
| Composite pesticide variable encompassing organophosphates, organochlorines or lice  treatment | 64-17 | 31-6 | 23-7 | 0.46 | 15-5 | 12-8 | 0.31 | 13-6 | 30-11 | 0.19 | 0.009 | 0.058 | 0.063 |

Values for case and control are number unexposed-exposed. Persons who rate the exposure as “unsure” are excluded. For some assessed drugs there were too few values for comparison. *Ctrl* control.

**Table S3** Gulf-specific exposures in first vs second test-set

| Exposure | All | 1st test-set | 2nd test-set | *P* for difference |
| --- | --- | --- | --- | --- |
| NBC suits | 4-35 | 1-19 | 3-16 | 0.27 |
| Wore a flea collar | 34-2 | 16-1 | 18-1 | 0.94 |
| Wore a uniform treated with pesticides | 17-12 | 8-8 | 9-4 | 0.30 |
| Wore permethrin-impregnated uniforms | 17-12 | 5-11 | 12-1 | 0.001 |
| Chemical or nerve gas attack | 12-13 | 5-9 | 7-4 | 0.17 |
| Diesel or petrochemical fuel on skin | 16-21 | 8-12 | 8-9 | 0.67 |
| Diesel or petrochemical fumes | 7-33 | 1-19 | 6-14 | 0.037 |
| Exhaust from heaters or generators | 14-21 | 5-14 | 9-7 | 0.072 |
| Fumes from munitions | 11-19 | 6-12 | 5-7 | 0.64 |
| Inhaled smoke from oil-well fires | 4-33 | 2-18 | 2-15 | 0.86 |
| Jet fuel burned in tent heaters | 25-11 | 12-7 | 13-4 | 0.39 |
| Mustard gas | 17-3 | 9-2 | 8-1 | 0.66 |
| Saw smoke from oil well fires | 4-35 | 2-18 | 2-17 | 0.96 |
| Received one or more immunizations in the  arm while in theater | 6-31 | 2-16 | 4-15 | 0.41 |
| Received one or more immunizations in the  buttocks while in theater | 15-19 | 7-10 | 8-9 | 0.73 |
| Heard chemical alarms sounded | 7-33 | 2-18 | 5-15 | 0.21 |
| Drank contaminated water | 19-8 | 8-5 | 11-3 | 0.33 |
| Drank diet soda | 26-10 | 12-6 | 14-4 | 0.46 |
| Drank water from a desert bag | 17-21 | 8-10 | 9-11 | 0.97 |
| Antimalarial drugs | 14-14 | 5-8 | 9-6 | 0.26 |
| Took ciprofloxacin | 18-8 | 5-6 | 13-2 | 0.024 |
| Took doxycycline | 20-7 | 7-4 | 13-3 | 0.31 |
| Took pyridostigmine pills | 7-26 | 1-17 | 6-9 | 0.016 |
| Other paints or solvents | 22-12 | 9-9 | 13-3 | 0.057 |
| Resprayed vehicles | 26-10 | 13-5 | 13-5 | 1.0 |
| Used or came into contact with freshly applied CARC paint | 16-7 | 8-6 | 8-1 | 0.11 |
| Insect repellents | 8-27 | 3-17 | 5-10 | 0.20 |
| Person pesticides | 20-16 | 8-12 | 12-4 | 0.036 |
| Pesticides handling | 24-13 | 11-9 | 13-4 | 0.17 |
| Pesticides on clothing or bedding | 15-15 | 6-11 | 9-4 | 0.065 |
| Saw the area in which you lived fogged or  sprayed with pesticides | 19-17 | 6-12 | 13-5 | 0.019 |
| Sprayed quarters with pesticides | 21-10 | 10-6 | 11-4 | 0.52 |
| Used pesticide cream or spray on skin | 15-23 | 6-14 | 9-9 | 0.21 |
| Bathed in local pond or river or Gulf waters | 32-9 | 13-7 | 19-2 | 0.049 |
| Burning rubbish or feces | 19-20 | 7-12 | 12-8 | 0.15 |
| Sandstorms | 4-36 | 2-18 | 2-18 | 1.0 |
| Used gas masks | 4-36 | 1-19 | 3-17 | 0.29 |
| Water treatment chemicals | 13-14 | 4-10 | 9-4 | 0.035 |
| Anthrax | 5-29 | 1-15 | 4-14 | 0.19 |
| Botulinum toxoid or “BT” vaccine | 14-4 | 5-4 | 9-0 | 0.023 |
| Cholera vaccine | 11-10 | 4-10 | 7-0 | 0.002 |
| Hepatitis A, B vaccine | 8-13 | 2-9 | 6-4 | 0.049 |
| Immunoglobulin vaccine | 7-21 | 3-11 | 4-10 | 0.66 |
| Meningococcal vaccine | 9-9 | 3-6 | 6-3 | 0.16 |
| Pertussis vaccine | 11-7 | 3-5 | 8-2 | 0.066 |
| Plague vaccine | 10-11 | 3-9 | 7-2 | 0.017 |
| Poliomyelitis vaccine | 12-10 | 4-7 | 8-3 | 0.087 |
| Tetanus vaccine | 4-25 | 1-14 | 3-11 | 0.25 |
| Typhoid vaccine | 9-17 | 3-13 | 6-4 | 0.031 |
| Yellow fever vaccine | 11-18 | 3-13 | 8-5 | 0.018 |

Values are number unexposed-exposed. “Unsure” about exposure were excluded from analysis. *NBC* nuclear biological chemical

**Table S4** Exposure predictors of MDA – exposures significant on univariable analysis, in the total sample

| Exposure | All  *n =* 81^*^ | | All case  *n* = 41 |  | All control  *n* = 40 | | 1st test-set  *n* = 40^*^ | | 2nd test-set  *n* = 41^*^ | |
| --- | --- | --- | --- | --- | --- | --- | --- | --- | --- | --- |
|  | *β (SE)* | *P* | *β (SE)* | *P* | *β (SE)* | *P* | *β (SE)* | *P* | *β (SE)* | *P* |
| Petroleum products (e.g., oil) | -6.0 (2.0) | 0.004 | -7.6 (2.4) | 0.003 | -2.0 (3.9) | 0.61 | -2.7 (1.8) | 0.14 | -7.6 (4.2) | 0.080 |
| Solvents | -4.5 (2.1) | 0.037 | -2.0 (3.0) | 0.50 | -9.9 (1.7) | <0.001 | -2.3 (1.6) | 0.18 | -6.2 (3.6) | 0.096 |
| Stored fuels | -6.6 (2.1) | 0.003 | -5.3 (2.7) | 0.059 | -8.0 (1.4) | <0.001 | -4.1 (1.7) | 0.019 | -5.8 (4.6) | 0.22 |
| Organophosphate  insecticides | -6.4 (2.3) | 0.007 | -5.2 (4.0) | 0.21 | -8.6 (1.5) | <0.001 | -2.9 (1.4) | 0.051 | +2.1 (2.8) | 0.45 |
| Organochlorines | -7.7 (1.4) | <0.001 | -5.8 (3.3) | 0.094 | -8.9 (1.9) | <0.001 | -2.4 (1.3) | 0.070 | -11 (2.5) | <0.001 |
| Lice treatment (e.g.,  Lindane) | -6.1 (1.6) | <0.001 | -5.2 (2.0) | 0.015 | -9.0 (1.7) | <0.001 | -2.6 (1.5) | 0.093 | -9.0 (2.4) | 0.001 |
| DEET | -6.1 (2.5) | 0.020 | -6.3 (3.7) | 0.095 | -6.5 (2.8) | 0.027 | -1.6 (1.4) | 0.25 | -5.8 (4.6) | 0.21 |
| Regular herbicide treatment at place of work | -7.5 (1.3) | <0.001 | -8.2 (1.7) | <0.001 | -7.5 (1.9) | <0.001 | -0.27(1.3) | 0.83 | -11 (2.4) | <0.001 |
| Selenium | -6.3 (3.0) | 0.038 | -6.1 (2.8) | 0.038 | dropped | - | 1.1 (1.8) | 0.53 | -8.4 (3.9) | 0.042 |
| BT vaccine received | -6.7 (2.2) | 0.004 | -5.2 (3.1) | 0.11 | dropped | - | -1.3 (1.4) | 0.36 | dropped | - |
| Hepatitis A vaccine | -4.8 (2.1) | 0.027 | -8.1 (3.0) | 0.012 | -0.89 (3.0) | 0.77 | -2.3 (1.9) | 0.23 | -3.3 (3.4) | 0.33 |
| Meningococcal vaccine | -6.5 (3.0) | 0.036 | -6.6 (3.1) | 0.043 | dropped | - | 0.45 (1.7) | 0.80 | -5.1 (5.9) | 0.40 |
| Olanzapine (Zyprexa) taken or prescribed | +10 (1.7) | <0.001 | +10 (1.8) | <0.001 | dropped | dropped | dropped | - | 4.7 (2.1) | 0.030 |
| Quetiapine (Seroquel)  taken or prescribed | +8.3 (1.6) | <0.001 | +8.5 (1.7) | <0.001 | dropped | dropped | dropped | - | 2.9 (2.1) | 0.17 |

Shown are values for those exposures that were significant predictors in the total sample and at least one subgroup (same direction). Regression with robust standard errors, adjusted for time-to-test, and ln(time-to-test). By case status are adjusted for test-set status. Assessments stratified by test-set are adjusted for case status. Variables are shown that are significant in the total sample adjusted for case status; and least borderline significant in at least one additional category. Nonsignificant values are shown for these variables in additional categories if these categories appear to contribute – if the absolute value of the coefficient exceeds 5 in the direction of effect in the total sample with P≤0.22. *** adjusted for case, *-* no data, *BT* botulinum toxoid, *SE* standard error.

**Table S5** Gulf-specific exposure predictors of MDA – exposures significant on univariable analysis (variables assessed for cases only, *n* = 41) adjustments

| Exposure | Time-to-test, ln (time-to- test) | | Time-to-test, ln (time-to- test), test-set | |
| --- | --- | --- | --- | --- |
|  | β (*SE*) | *P* | β (*SE*) | *P* |
| NBC suits | -7.4 (3.1) | 0.022 | -6.6 (2.8) | 0.025 |
| Chemical alarms | -6.1 (2.7) | 0.032 | -4.2 (2.2) | 0.064 |
| Chemical attack | -6.5 (3.7) | 0.083 | -4.1 (2.2) | 0.071 |
| Gas mask | -12 (3.8) | 0.004 | -6.1 (2.8) | 0.036 |
| Saw smoke | +10 (3.5) | 0.007 | +6.3 (3.1) | 0.053 |

Regression with robust standard errors. Adjusted for time-to-test, ln(time-to-test), Second set of coefficients also adjusts for test-set. All negative predictors related to potential chemical attack. Permethrin-impregnated uniforms was also a significant predictor (*P* = 0.011) on analysis not adjusted for test-set, but no relationship was preserved in test-set-adjusted analysis. *NBC* nuclear biological chemical; *SE* standard error.

**Table S6** Optimized MDA model for cases (*n* = 41)

| Exposure | Adjusted time, ln (time), first set*^a^* | | Adjusted time, ln (time) not first set | | Not adjusted time, ln (time) or first set | | Mean value of that variable in first set, second set*^b^* | | |
| --- | --- | --- | --- | --- | --- | --- | --- | --- | --- |
|  | β (*SE*) | *P* | β (*SE*) | *P* | β (*SE*) | *P* | 1st half mean *n*=20 | 2nd half mean *n*=21 | *P* for difference |
| Chemical attack | -6.5 (2.1) | 0.003 | -9.2 (3.3) | 0.009 | -9.1 (3.0) | 0.004 | 0.60 (0.42) | 0.43 0.36 | 0.17 |
| Composite pesticide | -6.3 (1.9) | 0.003 | -7.4 (2.6) | 0.009 | -7.6 (2.3) | 0.002 | 0.40 (0.50) | 0.14 (0.36) | 0.06*^c^* |
| 2GA | +5.9 (2.3) | 0.015 | +8.7 (3.3) | 0.013 | +8.5 (2.9) | 0.006 | 0.05 (0.15) | 0.19 (0.37) | 0.12 |

*^a^* First set refers to the first 20 matched pairs sent for testing. *^b^* Second set refers to the second 20 matched pairs sent for testing. *^c^* Regression with robust standard errors: *P =* 0.010. *R^2^ =* 0.72 (over adjusted), 0.39, 0.37. The first set has lower MDA values, but also materially greater exposures, so these cannot be presumed to be batch effects. In the fully adjusted model, if 2GA use is omitted among predictors, beta (*SE*) *P* are -5.9 (2.2) 0.012 for Chem. Attack; -6.5 (1.8) 0.001 for pesticide composite exposure. In the minimally adjusted model, these are -8.7 (3.2)0.010; -8.5 (2.2) 0.001. This two-predictor model produced an R^2^ of 0.29. *2GA* 2nd generation antipsychotic: olanzapine, quetiapine, risperidone (These are prescribed for many reasons that do not involve psychosis).

**Table S7** MDA – multivariable prediction in controls

| **Exposure** | **β (*SE*)** | ***P*** |
| --- | --- | --- |
| Model includes organochlorines and lice treatment |  |  |
| Organochlorines | -9.7 (1.9) | < 0.001 |
| Lice treatment | -9.8 (1.8) | < 0.001 |
| R^2^ | 0.31 |  |
| Model includes organophosphates-insecticides, organochlorines, lice treatment |  |  |
| Organophosphates- insecticides | -5.4 (2.6) | 0.046 |
| Organochlorines | -6.2 (3.0) | 0.049 |
| Lice treatment | -9.9 (1.9) | < 0.001 |
| R^2^ | 0.33 |  |
| Model includes solvents and lice treatment |  |  |
| Solvents | -9.0 (1.7) | < 0.001 |
| Lice treatment | -5.8 (2.7) | 0.039 |
| R^2^ | 0.33 |  |

In this two variable model (with full adjustments for time-to-test, ln(time-to-test), test set – but without the antipsychotic variable), coefficients are identical and p-values significant with or without exclusion of those who cite their exposure as “unsure” (coded as 0.5) – but the sample size is reduced to 25 if those citing exposure as “unsure” are excluded. To better support the larger number of adjustment variables, models shown include those who rated the variable as “unsure” (coded as 0.5). If those rating an exposure as “unsure” in the 3-variable model are excluded, despite the drop in sample size, each predictor remains significant.

Citations support increases in oxidative stress in animal models of GWI [3-7].

**References**

1. Faul F, Erdfelder E, Buchner A, Lang AG. Statistical power analyses using G*Power 3.1: tests for correlation and regression analyses. Behav Res Methods. 2009;41(4):1149-60.

2. Faul F, Erdfelder E, Lang AG, Buchner A. G*Power 3: a flexible statistical power analysis program for the social, behavioral, and biomedical sciences. Behav Res Methods. 2007;39(2):175-91.

3. Dickey B, Madhu LN, Shetty AK. Gulf War illness: mechanisms underlying brain dysfunction and promising therapeutic strategies. Pharmacol Ther. 2021;220:107716. doi: 10.1016/j.pharmthera.2020.107716

4. Kimono D, Sarkar S, Albadrani M, Seth R, Bose D, Mondal A, Li Y, Kar AN, Nagarkatti M, Nagarkatti P, et al. Dysbiosis-associated enteric glial cell immune-activation and redox imbalance modulate tight junction protein expression in Gulf War illness pathology. Front Physiol. 2019;10:1229. doi: 10.3389/fphys.2019.01229.

5. Kodali M, Hattiangady B, Shetty GA, Bates A, Shuai B, Shetty AK. Curcumin treatment leads to better cognitive and mood function in a model of Gulf War illness with enhanced neurogenesis, and alleviation of inflammation and mitochondrial dysfunction in the hippocampus. Brain Behav Immun. 2018;69:499-514. doi: 10.1016/j.bbi.2018.01.009

6. Shetty AK, Attaluri S, Kodali M, Shuai B, Shetty GA, Upadhya D, Hattiangady B, Madhu LN, Upadhya R, Bates A, et al. Monosodium luminol reinstates redox homeostasis, improves cognition, mood and neurogenesis, and alleviates neuro- and systemic inflammation in a model of Gulf War illness. Redox Biol. 2020;28:101389. doi: 10.1016/j.redox.2019.101389

7. Shetty GA, Hattiangady B, Upadhya D, Bates A, Attaluri S, Shuai B, Kodali M, Shetty AK. Chronic oxidative stress, mitochondrial dysfunction, Nrf2 activation and inflammation in the hippocampus accompany heightened systemic inflammation and oxidative stress in an animal model of Gulf War illness. Front Mol Neurosci. 2017;10:182. doi: 10.3389/fnmol.2017.00182.
